# Potyviral Helper-Component Protease: Multifaced Functions and Interactions with Host Proteins

**DOI:** 10.3390/plants13091236

**Published:** 2024-04-29

**Authors:** Veronika Hýsková, Kateřina Bělonožníková, Josef Chmelík, Hana Hoffmeisterová, Noemi Čeřovská, Tomáš Moravec, Helena Ryšlavá

**Affiliations:** 1 Department of Biochemistry, Faculty of Science, Charles University, Hlavova 2030, 128 43 Prague, Czech Republic; veronika.hyskova@natur.cuni.cz (V.H.); katerina.belonoznikova@natur.cuni.cz (K.B.); or chmelik@biomed.cas.cz (J.C.); 2 Institute of Microbiology of the Czech Academy of Sciences, Vídeňská 1083, 142 20 Prague, Czech Republic; 3 Institute of Experimental Botany of the Czech Academy of Sciences, Rozvojová 263, 165 02 Prague, Czech Republic; hoffmeisterova@ueb.cas.cz (H.H.); cerovska@ueb.cas.cz (N.Č.); moravec@ueb.cas.cz (T.M.)

**Keywords:** Potyvirus, viral protease, HC-Pro, interactions, host protein, gene silencing

## Abstract

The best-characterized functional motifs of the potyviral Helper-Component protease (HC-Pro) responding for aphid transmission, RNA silencing suppression, movement, symptom development, and replication are gathered in this review. The potential cellular protein targets of plant virus proteases remain largely unknown despite their multifunctionality. The HC-Pro catalytic domain, as a cysteine protease, autoproteolytically cleaves the potyviral polyproteins in the sequence motif YXVG/G and is not expected to act on host targets; however, 146 plant proteins in the *Viridiplantae* clade containing this motif were searched in the UniProtKB database and are discussed. On the other hand, more than 20 interactions within the entire HC-Pro structure are known. Most of these interactions with host targets (such as the 20S proteasome, methyltransferase, transcription factor eIF4E, and microtubule-associated protein HIP2) modulate the cellular environments for the benefit of virus accumulation or contribute to symptom severity (interactions with MinD, Rubisco, ferredoxin) or participate in the suppression of RNA silencing (host protein VARICOSE, calmodulin-like protein). On the contrary, the interaction of HC-Pro with triacylglycerol lipase, calreticulin, and violaxanthin deepoxidase seems to be beneficial for the host plant. The strength of these interactions between HC-Pro and the corresponding host protein vary with the plant species. Therefore, these interactions may explain the species-specific sensitivity to potyviruses.

## 1. Introduction

Virus-encoded proteases are the smallest proteases involved in the life cycle of both plant and animal viruses, including potato virus Y (PVY), human immunodeficiency virus (HIV), severe acute respiratory syndrome coronavirus 2 (SARS-CoV-2), or hepatitis C virus (HCV) [1,2,3]. A mechanism of action of these vertebrate viruses involves the proteolytic cleavage of host proteins, thereby inhibiting their activity [4], e.g., in innate immune system evasion [5,6]. Alternatively, some protein fragments released by proteases benefit the virus infection cycle [4]. As shown by protease degradomics (i.e., summaries of protease substrates and products), the HIV-1 protease cleaves over 120 host proteins [7]. In turn, the poliovirus 3C protease [8] and Zika virus protease [9] cleave 72 and 31 host proteins, respectively. 

Almost half of all plant viruses encode at least one protease, but whether they employ this strategy remains unknown [4]. Many plant viruses express their proteins through a polyprotein strategy; so, to regulate the release of functional, mature proteins and/or intermediate polyproteins, they must contain protease domains [10]. Plant viral proteases stand out for their multifunctionality, participating in horizontal virus transmission and suppressing plant defense responses, among other functions [11]. 

In this context, this review aimed to compile the amino acid sequences or motifs responsible for specific HC-Pro functions across potyvirus species and to gather the up-to-date known interaction partners of HC-Pro in host cells, discussing the implications of such interactions. In addition, we analyzed putative host proteins carrying an HC-Pro cleavage sequence. As such, this review may help us to better understand the role of proteases in infected plant host cells.

## 2. Potyvirus Genome Organization

Together with seven other genera, potyviruses form one of the largest families of plant viruses with a broad host range and geographical distribution, known as *Potyviridae*. The genus Potyvirus encompasses 167 species of plant viruses transmitted by aphids in a non-persistent mode [12,13,14]. Although the host range of most potyvirus species can be narrow, their economic importance is considerable given that there is a pathogenic potyvirus for almost every cultivated crop, including potatoes, sugarcane, soybeans, zucchinis, turnips, tobacco, plums, and watermelons [12,15,16]. 

Potyviruses consist of flexuous, filamentous particles of 700–750 nm in length [14] carrying a positive-sense, single-stranded RNA (+ssRNA) with a genome of approximately 9.3–11 kb in length, encoding 11 viral proteins [14,17]. Polyprotein processing enables a controlled and timely release of mature functional gene products or partly processed intermediate polypeptides with various biological activities. Thus, viruses have also developed various mechanisms to regulate the activity of viral proteases and/or the efficiency of polypeptide cleavage at specific sites [10]. 

**Figure 1:**
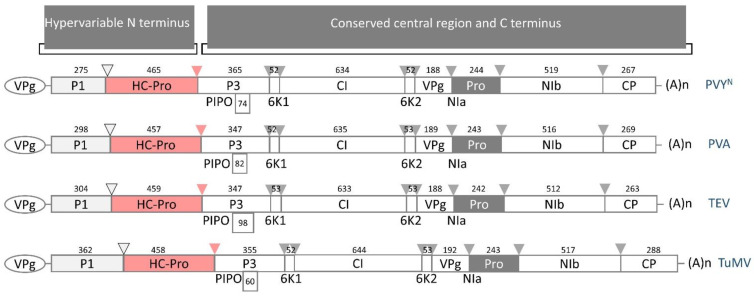
Schematic diagram of the genomic organization of four potyviruses, which encode 11 putative proteins. Potyvirus 1 (P1) is involved in the translation and modulation of replication. The multifunctional Helper-Component protease (HC-Pro) is discussed in this review. Potyvirus 3 (P3) participates in virus replication. The N-terminal half of P3, fused to the Pretty Interesting Potyvirus open reading frame (P3N/PIPO), facilitates cell-to-cell movement. The 6-kiloDalton protein 1 (6K1) forms replication vesicles. Cylindrical Inclusion (CI) functions as an RNA helicase. 6K2 is the 6-kiloDalton protein 2. The viral genome-linked protein (VPg) is involved in translation, virus movement, and replication. The Nuclear Inclusion a protease (NIa-Pro) cleaves polyproteins at seven sites. Nuclear Inclusion b (NIb) is an RNA-dependent RNA polymerase. The coat protein (CP) participates in virus movement, virion formation, and aphid transmission. Across the *Potyviridae* family, the N-terminal regions are defined as hypervariable termini due to differences in protein organization and size, whereas regions from P3 to CP are relatively conserved, with some exceptions, as mentioned above [14]. The number above each box expresses the size of the amino acid sequence of the corresponding protein (as presented in [18]). Cleavage sites are indicated with arrows (HC-Pro: red, P1: light gray, NIa: gray). PVY^N^—necrotic strain of potato virus Y (GenBank Accession No.: UVJ64998), PVA—potato virus A (GenBank Accession No.: QYA72248), TEV—tobacco etch virus (GenBank Accession No.: UTQ11696), TuMV—turnip mosaic virus (GenBank Accession No.: WIM35122).

Upon viral genome translation, the large polyprotein is proteolytically processed by three specific viral proteases, namely the Potyvirus 1 protease (P1), the Helper-Component protease (HC-Pro), and the Nuclear Inclusion a protease (NIa-Pro). Even though the potyviral genome is under strong negative selection, the genome and polyprotein sequence are remarkably stable [14]. The N-terminal polyprotein regions (upstream of the P3 protein-coding sequence) are defined as hypervariable because they markedly differ in organization and size, especially when considering the whole *Potyviridiae* family. In contrast, the central and carboxy regions (spanning from P3 to the coat protein, CP) of the remaining mature proteins are relatively conserved in composition and size and thus are defined as conserved (Figure 1 [18]). Moreover, the host range has been correlated with the frequency of sites under positive selection; hypervariable areas map to the N-terminal region of P1 and CP, the N- and C-terminal regions of HC-Pro, the C-terminal region of P3, the viral genome-linked protein (Vpg), and the nuclear-inclusion protein b (NIb), in addition to the sequence of NIa protease cleavage sites [14]. Potyviral NIa processes the polyprotein at seven cleavage sites, yielding mature proteins such as CP and Nib, which is the RNA-dependent RNA polymerase responsible for viral genome replication [4,18,19]. Located at the very amino terminus of the potyviral polyprotein, the P1 protease and HC-Pro, in turn, cleave the polyprotein at a single site for autocatalytic release. Because HC-Pro and P1 function autoproteolytically, it is assumed that they most likely do not process host proteins. 

HC-Pro can be divided into N-terminal (1–100 amino acids, AAs), central (101–299 AAs), and C-terminal (300–459 AAs) domains with proteolytic activity [20]. As a leader protease, HC-Pro plays a key role in the viral life cycle. This protease is required for efficient genome amplification, RNA silencing (antiviral plant defense) suppression, virus transport inside infected plants, and aphid transmission [20,21]. These functions are driven by protein domains different from the proteolytic domain [4]. The HC-Pro C-terminal region belongs to the cysteine protease structural domain (CPD) and is not homologous to any region of cysteine proteases in cells and viruses other than those of the potyvirus family [22]. The molecular weight of HC-Pro is approximately 50 kDa. The biologically active transmissible forms of HC-Pro are dimers (approximately 100 kDa) and trimers (approximately 150 kDa) [23,24,25].

## 3. HC-Pro Is a Multifunctional Protein

HC-Pro was first identified as an indispensable helper factor for aphid transmission, even for non-transmissible potyviruses [11]. The transmission function involves two conserved motifs, namely an N-terminal KITC motif (AAs 52–55, numbered according to the lettuce mosaic virus, LMV) and a PTK motif (AAs 310–312), which likely enables capsid binding [23]. Other regions of the N-terminal domain were later proposed to affect HC-Pro transmissibility [26,27,28,29], confirming the compatibility of different HC-Pro proteins in supporting the transmission of other potyviruses (trans-complementation) as an important helper strategy for non-persistent virus transmission [26]. 

**Figure 2:**
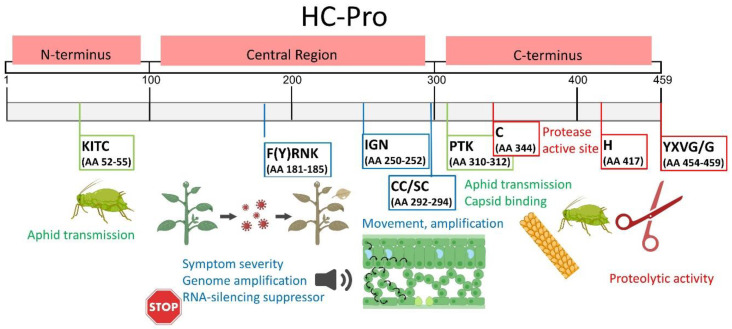
The structure and the best-characterized functional regions of potyviral HC-Pro. The HC-Pro structure can be divided into three domains. The N-terminal domain (AAs 1–100) is involved in aphid transmission. The central domain (AAs 101–299) participates in RNA silencing suppression, genome amplification, symptom severity, and viral movement. The C-terminal domain (AAs 300–459) displays proteolytic activity and is thus also known as the cysteine protease structural domain (CPD). The best-characterized functional motifs (numbered according to LMV, UniProtKB database sequence P89876 · POLG_LMVE) are shown in color-coded frames. In green, KITC and PTK are required for aphid transmission. In blue, the motif F(Y)RNK is associated with symptom severity, genome amplification, virus movement, RNA silencing suppression, and si-RNA-binding activity; the IGN motif is associated with genome amplification; and the CC/SC motif is associated with long-distance transport. In red, the YXVG/G motif is required for protease activity. This scheme was modified from [23,26].

Most HC-Pro functions are provided by the central domain in which the F(Y)RNK box is a highly conserved motif located at AAs 181–185 and participates in multiple functions, including symptom severity, genome amplification, virus movement, RNA silencing suppression, and siRNA-binding activity. The IGN (AAs 250–252) motif is crucial for genome amplification; the CC/SC (AAs 292–294) motif is needed for long-distance transport; and the C-terminal GYCY motif, in the active center (C344 in Figure 2), is crucial for cysteine protease activity [20]. Conserved cysteine residues in the N-terminal domain of HC-Pro form a secondary zinc-finger structure, which may be responsible for binding metal ions and nucleic acids [30]. The ability of HC-Pro to bind to nucleic acids has been experimentally shown via oligo(dT) purification [30,31]. More recently, HC-Pro was found to preferentially bind to viral sRNAs with 21–22 nucleotides containing 5′-terminal adenines, thereby suppressing silencing [32].

### 3.1. HC-Pro Suppresses RNA Silencing

The potyvirus fusion protein P1/HC-Pro was the first viral RNA silencing suppressor to be identified, with early studies on its mechanism showing that P1 enhances the suppressive effect of HC-Pro on RNA silencing [33,34]. Also known as RNA interference (RNAi), RNA silencing is a mechanism of innate immunity. This mechanism relies on the sequence-specific suppression of gene expression through transcriptional and translational repression induced by double-stranded RNA (dsRNA), an intermediate that is produced during viral replication [35]. By activating ribonuclease dicer enzymes, exogenous dsRNA initiates the production of 21–24 bp long virus-derived short-interfering RNAs (vsiRNAs). 

vsiRNAs are stabilized by the HUA enhancer 1 (HEN1)-mediated methylation of their 3’-ends. Once the 3’-end is methylated, the vsiRNA is then converted into single-stranded RNA when the complementary-sense RNA strand is degraded by Argonaute (AGO) enzymes. Subsequently, the anti-sense RNA is incorporated into the RNA-induced silencing complex (RISC), which binds to the target sense sequence via the complementary anti-sense RNA. Using this strand, the RISC further binds to and degrades additional copies of the complementary-sense RNA [26,35]. 

HC-Pro can counteract this plant defense barrier by targeting multiple steps of the aforementioned cascade. Most potyviral HC-Pros suppress host RNA silencing by directly binding to and sequestering vsiRNAs in a size-specific manner [26,36]. Alternatively, HC-Pro can interfere with vsiRNA methylation (More details in Section 4.4) [37,38,39], AGO-containing effector complexes [3], and RNA-dependent RNA polymerases [26]. 

Although, comparisons of the suppressive ability of HC-Pros from different viruses are limited by their different and/or narrow host ranges. They differ in their ability to inhibit the RISC and degrade AGO1, as shown by genetic transformation. For example, TuMV-encoded HC-Pro more efficiently triggers AGO1 degradation by autophagy, unlike zucchini yellow mosaic (ZYMV) and tobacco etch (TEV) virus HC-Pros [33]. Moreover, the TuMV HC-Pro specifically inhibits HEN1 activity. As a result, unmethylated microRNAs accumulate in the cytoplasm without being loaded into AGO1 [40]. 

HC-Pro may interact with bridging host factors, which in turn may also interact with AGO1 via the motif WG (W208, G209), an amino acid pair of HC-Pro [33]. HC-Pro from PVA and the host protein VARICOSE (VCS) are important for RNA silencing suppression, as discussed below in more detail [41]. HC-Pro targets have been identified in the antiviral RNA-silencing pathway and cascade steps, where potyviral HC-Pro may block this defense response, as has been excellently illustrated in the review by Valli et al., 2018 [26]. While mutations that inactivate the proteolytic activity of HC-Pro have no effect on RNA silencing suppression, the proteinase domain itself may still be required for suppression [42]. 

**Figure 3:**
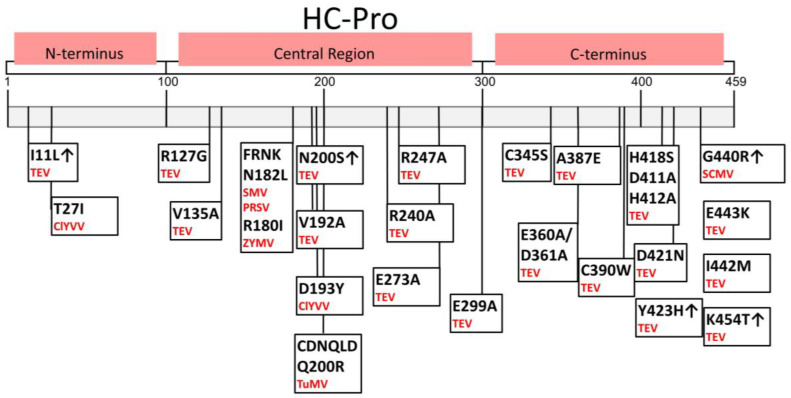
Amino acid sequences of HC-Pro crucial for gene silencing suppression and symptom development, identified using HC-Pro mutants (changed AAs and positions are indicated). Particular viruses are in red; ↑ indicates increased suppressor activity; no symbol indicates hypo- or null suppression activity after a particular change [20,42,46,47,48,49,50,51,52].

Several studies on AA substitutions in the HC-Pro sequence have found some AAs to be critical for silencing suppression in all HC-Pro regions; some examples of key HC-Pro AAs in silencing suppression are shown in Figure 3. Moreover, it was shown that transgenic tobacco plants expressing an intron-containing hairpin RNAi constructed with a 201 fragment of the papaya ringspot virus (PRSV) HC-Pro gene exhibited complete resistance to PRSV [43]. In potato plants (cv. Agria), applying a combination of ds/si RNAs specific to CI-6K2-NIa and HC-Pro regions, controlled PVY^N/NTN^ infection [44]. These findings demonstrate that identifying HC-Pro motifs required for gene silencing suppression could help use hairpin RNA-induced silencing, which is such a powerful tool for developing plant viral resistance by silencing specific viral RNAs [45].

Interestingly, in areca palm necrotic spindle-spot virus (ANSSV), a newly identified member of the *Potyviridae* family, more specifically of the genus Arepavirus, two closely related cysteine proteases HC-Pro1 and HC-Pro2 were identified in tandem at the N-terminal region [36]. These two HC-Pros might have evolved contrasting RNA silencing suppression activities and function in a coordinated manner to maintain viral infectivity. HC-Pro2 exhibits RNA silencing suppression activity in both the N-terminal and C-terminal regions, whereas the N-RNA-silencing activity of the terminal region of HC-Pro1 is suppressed by its C-terminal protease domain [36]. The autocatalytic cleavage sites of HC-Pro1 and HC-Pro2 are located at positions 273G/S274 and 574G/G575, respectively [36].

### 3.2. HC-Pro Presence Directly Correlates with Symptom Severity, Pathogenicity, and Virus Accumulation

During potyviral infection, most host plants show typical symptoms, especially chlorosis, chlorotic local lesions, malformations, mosaic, stunting, or vein clearing within 1–2 weeks from the onset of infection, which may be related to temperature and heat-shock protein recruitment [53,54,55]. Symptom severity and the effectiveness of HC-Pro’s suppression of silencing are closely related, as shown in almost all studies mentioned in Section 3.1 and Figure 3. For example, clover yellow vein virus (ClYVV) HC-Pro is involved in necrotic symptom development in *Vicia faba* [51]. Furthermore, the PVY HC-Pro E419D mutant is unable to induce tuber necrosis in three potato cultivars susceptible to potato tuber necrosis ringspot disease caused by PVY isolates. As such, E419 may be crucial for PVY aggressiveness in tubers and is likely involved in other virus/host interactions [56].

In addition to E419, the residue N339 of HC-Pro is a PVY molecular determinant of vein necrosis induction in tobacco [57]. A single amino acid substitution in HC-Pro can affect both symptom severity and the RNA silencing suppression efficiency [52]. For instance, symptom severity and virus accumulation can be altered by a single amino acid change in the HC-Pro sequence of the plum pox virus (PPV) [58,59]. Broadly speaking, the more effective the RNA silencing suppression is, the more severe the plant symptoms will be, and vice versa. 

Studies have also assessed the effects of disrupting the function of highly conserved motifs FRNK, CDNQLD, and even KITC on symptom severity. When substituting I for R in the FRNK motif of the zucchini yellow mosaic virus (ZYMV), severe infection symptoms became mild in squash plants and disappeared in cucumber, melon, and watermelon plants [49,60]. The same trend was observed in sugarcane infected with sugarcane mosaic virus (SCMV) with a point mutation in the FRNK motif, showing reduced virulence and virus accumulation [50,61,62,63,64]. In a mild isolate of ZYMV, a natural mutation was found in the conserved CDNQLD motif. This D-to-Y point mutation in the CDN markedly reduced symptoms in the host plants. Based on the localization of CDNQLD near the FRNK motif, these two motifs may be part of a larger conserved potyviral sequence that influences symptomatology (Desbiez et al., 2010). 

The direct effect of the aforementioned sequences on symptoms may be caused by their involvement in RNA silencing, in HC-Pro interactions with proteasomes, or in the disruption of chloroplast function through interactions with the chloroplast division-related factor MinD or with ferredoxin-5 [61,62,63,64,65]. HC-Pro also enhances viral titer/particle yield by stabilizing its cognate coat protein, positively affecting the yield of virions and, consequently, improving the infectivity of viral progenies [13]. *Nicotiana tabacum* plants overexpressing the chilli veinal mottle virus (ChiVMV) HC-Pro showed a higher susceptibility to tobacco mosaic virus (TMV) and cucumber mosaic virus (CMV) infection at the early stages, but a stronger tolerance at the later stages, than wild-type plants. HC-Pro affects heterologous virus infection through salicylic acid and auxin pathways [66].

### 3.3. Proteolytic Activity of HC-Pro

In protease-coding viruses, polyproteins are cleaved at the proper time and place to optimize virus production by orchestrating replication and maturation [67]. To the best of our knowledge, though, no study has clarified the order in which the three potyviral proteases (P1, HC-Pro, and NIa) catalyze polyprotein processing. Proteolytic activity controls the concentration of individual key viral proteins. Usually, protease activation is delayed until tethered proteins in the polyprotein reach the proper virion assembly site. Protease activity is also suppressed when uncleaved, assembled polyproteins are converted into a mature capsid, whereas proteases play a key role in mediating disassembly upon virus entry into a newly infected cell [67]. 

**Figure 4:**
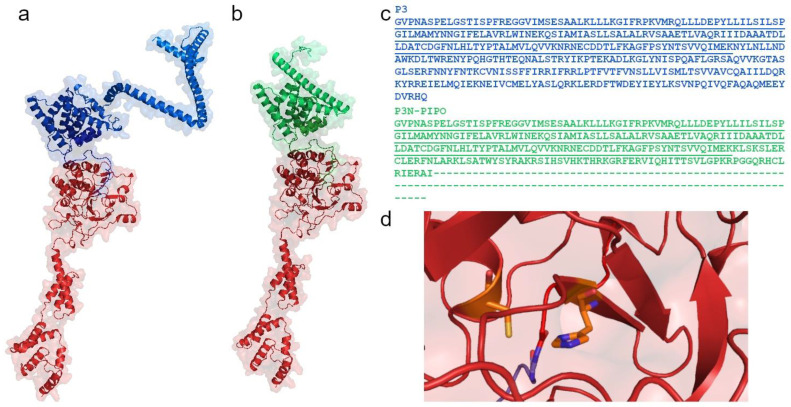
Structural model of the PVY HC-Pro (red) bound to either the P3 protein (blue, (**a**)) or P3N-PIPO (green, (**b**)). Characteristic structure of the HC-Pro active site, containing α1-α2-α3-β1-β2 regions. The protein sequence alignment of P3 and P3N-PIPO shows the same amino acids (underlined) (**c**). The proteins HC-Pro (red) and P3 (blue) are connected by a Gly–Gly bond, which is autocatalytically cleaved by HC-Pro. The catalytic Cys residue located at the N-terminus of the α-helix and the catalytic His residue in a β-strand form the active site, as shown in orange (**d**). This model of the HC-Pro, P3, and P3N-PIPO proteins was created using AlphaFold2 [73] in ColabFold [74]. The sequences were retrieved based on P0CJ93 from the database UniProtKB. The image was produced in PyMol [75].

The HC-Pro’s autoproteolytic activity between HC-Pro and P3 is essential for virus viability [68]. In addition to a conserved open reading frame (ORF), the genus Potyvirus has another ORF that produce a protein known as P3N/PIPO (the N-terminal half of P3 fused to the Pretty Interesting Potyvirus open reading frame). P3N-PIPO production requires an adenine insertion caused by RNA-dependent RNA-polymerase slippage at a conserved GAAAAAA sequence [69] (Figure 4).

Biochemical analyses of recombinant HC-Pro have confirmed that a large C-terminal domain is highly resistant to proteolysis [23]. In the active center of HC-Pro, one cysteine and one histidine form the catalytic dyad responsible for proteolysis. So, HC-Pro belongs to the cysteine-type proteinase family [26]. The crystal structure of the HC-Pro catalytic domain was first solved at 2.0 Å resolution by Guo et al. (2011), who found that the overall structure of the TuMV cysteine protease domain significantly differs from papain-like structures, especially in size (122 versus 212 AA residues) and in the lack of the third catalytic residue (Asn), as with the nsP2 protease of the Venezuelan equine encephalitis alphavirus. However, HC-Pro active sites share a similar configuration with the catalytic Cys 344 residue located at the N-terminus of the α-helix, and with the catalytic His 417 residue in a β-strand. As per standard convention, the five residues are named in the N-to-C order as P4, P3, P2, P1, and P1´, and their respective enzyme-binding sites are termed S4, S3, S2, S1, and S1´ [70]. 

In the TuMV HC-Pro, the C-terminal tetrapeptide (P1–P4) bound to the active-site cleft is formed by Gly 458, Val 457, Arg 456, and Tyr 455. This active site maintains a conserved catalytic cleft of papain-like proteases. In the active configuration, Cys 344 is deprotonated by His 417 and acts as nucleophile, attacking the carbonyl carbon of the scissile bond [68]. In PVY HC-Pro, Cys 342 and His 415 (numbered based on the HC-Pro sequence in UniProtKB: P0CJ93) are key AAs of the active site (Figure 2 and Figure 4). HC-Pro recognizes the consensus sequence YXVG/G and cleaves the Gly–Gly dipeptide at its own C-terminus, which then probably remains bound to the active center and prevents the proteolytic degradation of host proteins [68]. Some authors have suggested that the cleaved product binds to the active cleft, thereby preventing HC-Pro from targeting host proteins [68]. However, in vitro experiments with HC-Pro and proteins with a specific cleavage site as with potential substrates have not been performed yet. Therefore, we cannot draw conclusions about the enzyme kinetics of the HC-Pro catalyzed reaction and its possible product inhibition and its ir-or reversibility. 

Similar autoinhibitory structures were also predicted for the hepatitis C virus (HCV) NS2 protease, but this protease is catalytically active as a dimer with two composite active sites, i.e., the catalytic His 143 and Glu 163 residues are provided by one monomer and the third catalytic partner (Cys 184) is provided by the other monomer [71]. In addition, the HCV NS2 cysteine protease catalytic domain is regulated by the neighboring NS3 N-terminal domain [72]. Along these lines, a protein cofactor, the N-terminal region of protein P3 (in both P3 and P3N-PIPO), may also be involved in the catalytic activity of HC-Pro (Figure 4). To identify a substrate containing the sequence recognized by HC-Pro, we searched the YXVG/G sequence motif within *Viridiplantae* in the UniProtKB database using PROSITE. In total, 146 plant proteins may be cleaved by HC-Pro (Supplementary Table S1), and at least 14 of these proteins are involved in plant defense (Supplementary Table S2). In addition to plant defense (e.g., endoribonuclease dicer homolog 3b and ToMV-susceptible protein Tm-1), some proteins participate in photosynthesis (e.g., chlorophyll a–b-binding protein) and secondary metabolism (e.g., polyamine oxidase 6 and polyphenol oxidase E). Theoretically, protein cleavage may facilitate viral infection either directly, by disrupting the plant defense system, or indirectly, by altering plant metabolic pathways.

### 3.4. HC-Pro Is Involved in Aphid Transmission

Potyviruses can be rapidly and non-persistently inoculated into plants by more than 200 aphid species, the most common vectors of plant viruses [14,76]. Because they are non-persistently transmitted, plant virions are not translocated to the hemolymph; instead, they are only retained for a short period in the insect stylet and are lost once the insect molts [77]. During transmission, HC-Pro binds to the aphid receptor on the stylet and to virus particles through the DAG motif (or related variants) of the viral CP [12,77]. 

Among potyviruses, the HC-Pro sequence contains several highly conserved regions, some of which are crucial for aphid transmission. A highly conserved DAG motif in the CP directly interacts with a PTK motif located in the C-terminus of HC-Pro, facilitating HC-Pro binding to CP virions. Moreover, the KITC motif and its functionally equivalent motifs in the N-terminus of HC-Pro appear to be critical for virus retention in the stylets (Figure 2) [12,77]. According to the “bridge hypothesis” or “helper strategy”, HC-Pro acts as a reversible link (molecular bridge) between the CP of non-persistent potyviruses and insect vector receptors [12,26]. 

A characteristic feature of HC-Pro is also its trans-complementation, which prompted the discovery of HC-Pro itself. Trans-complementation enables viruses that cannot be transmitted by aphids in a single infection (such as Potexviruses) to become transmissible in a mixed infection when potyviral HC-Pro is additionally provided by potyviruses [77]. Different forms of HC-Pro allow the aphid transmission of potyviruses and a few other viruses from various families thanks to a domain with a DAG motif of the potexvirus (potato aucuba mosaic virus) CP, which facilitates virus transmission in the presence of the potyvirus (PVY) HC-Pro [12]. In addition, HC-Pro’s aphid stylet-binding specificity varies with the virus strain. While the PVY^NTN^ HC-Pro only facilitates the transmission of the homologous virus, the HC-Pro proteins of PVY^O^ and PVY^N:O^ facilitate the transmission of all tested strains (NTN, O, N:O). PVY^NTN^ predominantly binds to the aphid stylet over PVY^O^ when aphids feed on a mixture of these two viruses [78]. 

### 3.5. The Role of HC-Pro in Viral Transport

The role of HC-Pro in viral transport (short-distance transport from cell to cell and long-distance transport from leaf to leaf through the phloem) is not yet fully understood due to the different requirements for both types of movement and to the lack of conclusive genetic evidence. HC-Pro mutants that show intercellular movement-defective or systemic infection-defective phenotypes always exhibit reduced levels of viral genome replication. Accordingly, these movement-defective phenotypes may derive from incompetent viral replication [79].

Several amino acids with the CC/SC sequence motif (Figure 2) in the central region of HC-Pro have been implicated in long-distance movement and genome amplification [80,81]. However, more recent studies have shown that HC-Pro is correlated with RNA silencing suppression. For this reason, the role of the amino acid motif in transport may be misleading [26]. 

In plants, viruses are usually transported from cell to cell through one or more viral proteins with special movement functions [82,83]. Three potyviral proteins, namely P3N-PIPO, CI, and CP, are known to function in potyviral cell-to-cell movement [79]. As the only potyviral movement protein (MP), P3N-PIPO directs the Cylindrical Inclusion (CI) protein from the cytoplasm to the plasmodesmata, where CI forms conical structures for intercellular movement [82,84]. 

Three modes of transport have been described in potyvirus cell-to-cell movement. One of them is mediated by the second 6-kDa membrane-anchoring protein (6K2). Here, potyviruses use the host endomembrane system to produce membranous vesicles capable of moving between cells. In the second mode of transport, virions and ribonucleoprotein complexes are transported through plasmodesmata. The third mode of viral transport was proposed as the movement of extracellular viral vesicles in the apoplasm based on the observation of turnip mosaic virus (TuMV)-induced vesicles [84,85].

Potyviral CP and HC-Pro may be involved in intercellular movement because they (a) promote movement from cell to cell, (b) increase the plasmodesmal size exclusion limit, and (c) facilitate viral RNA movement from cell to cell [86]. HC-Pro also interacts with the microtubule-associated protein HC-Pro-interacting protein 2 (HIP2) of potato and tobacco plants during potyviral (PVA and PVY) infection, as discussed below [82,87]. HC-Pro participates in viral intercellular movement by stabilizing the CP, thereby increasing the yield of virions, and by being present (along with VPg) at the protruding tips at the ends of purified virions [79]. These tips act as a guiding device for the directional transport of modified virion complexes to and through plasmodesmata via interactions between CI and HC-Pro/VPg [88,89,90].

## 4. HC-Pro Interacts with Plant Host Proteins

**Figure 5:**
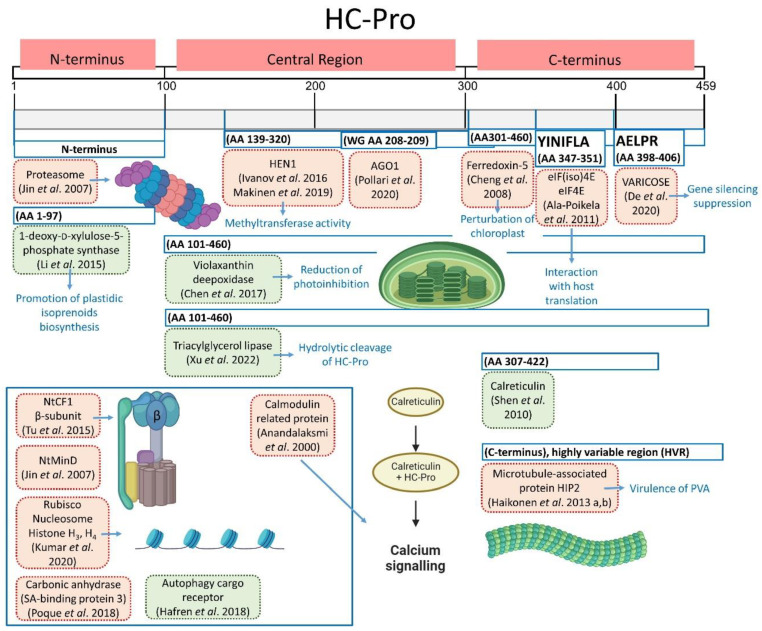
HC-Pro interactions with various host proteins. Interactions promoting virus infection are shown in red [3,37,41,62,63,64,87,91,99,101,102,103,104,105], whereas interactions favoring plant defense responses are shown in green [95,96,97,98,106]. Predicted HC-Pro binding sites are indicated by blue frames. The inset figure groups proteins whose binding sequences have not been determined yet or have only been identified in individual amino acids, as in the study by Kumar et al. 2020 [99].

The multifunctional viral protein HC-Pro is involved in many viral infection phases in plants and thus interacts with several other viral proteins (CP; RNA helicase, known as CI; P1; VPg; and NIa) and nucleic acids [91,92]. However, HC-Pro also interacts with various host proteins, which are important not only for promoting the life cycle of the virus but also for preventing plant defense responses [91,93]. HC-Pro interactors have been identified using various approaches, including yeast two-hybrid systems, bimolecular fluorescence complement assays, immunoprecipitation [63,64,91,94,95,96,97,98], affinity purification coupled with mass spectrometry [37,98], gel filtration, native electrophoresis, immunochemical analyses, and in silico protein–protein modeling [99,100]. Through specific binding sites, HC-Pro can interact with the enzymes, proteins, and molecular machinery of a host, such as ATP synthases, Rubisco, histones, proteasome, nucleosome, methionine cycle enzymes, and others (Table 1, Figure 5, with relevant references). The interaction partners of HC-Pro do not bind to the catalytically active site of the protease domain, but rather bind to other regions of the C- and N-terminal domains or central region of HC-Pro ([26,100], Table 1). In the following section, the identified HC-Pro regions and specific amino acid residues responsible for interactions regarding viral infection spread or plant defense responses are discussed. 

### 4.1. HC-Pro’s Interactions with the 20S Proteasome May Be an Alternative Pathway to RNA Silencing 

The 20S proteasome is a eukaryotic protein complex with a barrel-shaped structure in which the 20S core associates with a 19S regulatory complex, forming the 26S particle. The 26S proteasome degrades ubiquitinated proteins in an ATP-dependent fashion [61]. In addition to its proteolytic activity, the ubiquitin/26S proteasome system also exhibits ribonuclease (RNase) activity, degrading the genomic RNA of tobacco mosaic virus (TMV) and LMV RNAs in vitro [61,94]. More specifically, the recombinant *A. thaliana* proteasome α-5 subunit harbors RNase activity and hence can degrade TMV and LMV RNAs in vitro [94]. 

Furthermore, the potato virus Y (PVY) HC-Pro can interact with specific AA residues of three *Arabidopsis* 20S proteasome subunits (known as PAA, PBB, and PBE, or α-1, β-2, and β-5) [63]. The LMV HC-Pro can also bind to the proteasome and inhibit its 20S endonuclease activity in vitro, without affecting or only slightly stimulating its proteolytic activity [61]. These findings indicate that viruses modulate the proteasome-based protein degradation pathway for their own purposes, that is, to increase the turnover of host proteins involved in defense and/or virus proteins necessary in the earlier stages of its cycle [61,63]. In a manner reminiscent of RNA silencing suppression, HC-Pro inhibits proteasome 20S RNase activity [61], most likely to sustain high levels of viral RNA translation. 

Besides proteasome degradation, autophagy may also be involved in plant virus infection [106]. Selective autophagic degradation of the viral capsid protein and particles promotes antiviral immunity. The autophagy cargo receptor NBR1 suppresses turnip mosaic virus (TuMV) infection by targeting potyvirus-induced RNA granules with HC-Pro for degradation. However, potyviral proteins VPg and 6K2 can impair flux through the autophagy cargo receptor NBR1 and HC-Pro degradation, thereby enabling potyviruses to evade antiviral autophagy [106]. 

### 4.2. HC-Pro’s Interactions with Chloroplastic Proteins May Decrease Photosynthesis

The photosynthetic rate of virus-infected plants is always reduced. However, the mechanism underlying this phenomenon remains unclear [101]. HC-Pro may be a “general” pathogenicity factor [92] that accumulates in the chloroplasts of infected plants [101,113]. In *Nicotiana tabacum* L., the PVY HC-Pro interacts with MinD, a member of the ParA ATPase family, producing asymmetric chloroplast division in PVY-infected leaves. HC-Pro competes with MinE to bind to MinD, which may cause the formation of abnormal chloroplasts in PVY-infected plants [101]. HC-Pro also interacts with the ATP-synthase CF1 β-subunit, which plays a key role in reducing the photosynthetic rate of PVY-infected plants [107]. Moreover, the bean common mosaic virus (BCMV) HC-Pro interacts with a key enzyme of the Calvin cycle, Rubisco [99]. In turn, Rubisco interacts with other proteins encoded by potyviruses (such as the coat protein, P3, or the movement protein) [114,115]. HC-Pro specifically interacts with other chloroplastic proteins, such as maize ferredoxin-5. This interaction may disturb the post-translational import of ferredoxin-5 into maize bundle-sheath cell chloroplasts, potentially perturbing chloroplast structure and function [62].

### 4.3. HC-Pro’s Interactions with Host Transcription and Translation Factors May Increase Virulence and Suppress Gene Silencing

Potyviral HC-Pro alters the expression of various transcription factors and signaling pathways, controlling development and pathogen defense in plants [87]. Specific plant transcription factors can serve as host factors for plant defense suppression (gene silencing). Ethylene-inducible transcription factor RAV2/EDF2 (hereafter referred to as RAV2) is an example of an HC-Pro-interacting protein required for primary RNA silencing suppression, not only by potyvirus HC-Pro but also by carmovirus P38, the silencing suppressor from the virus family *Tombusviridae*, unrelated to potyviruses [108]. As confirmed by the hairpin transgene-silencing system, HC-Pro requires RAV2 to block the activity of primary siRNAs, whereas transitive silencing is suppressed independently of RAV2. RAV2 is also responsible for many HC-Pro-mediated morphological anomalies in transgenic plants [108].

Positive-strand RNA viruses are known to manipulate the activity and subcellular localization of translation initiation factors to gain access to the host translation machinery [91]. PVA, TEV, and PVY HC-Pro proteins interact with the cap-binding protein (the eukaryotic translation initiation factor eIF4E), particularly with the isoform eIF(iso)4E of tobacco and potatoes in planta. These interactions are controlled by an eIF4E-binding motif (YINIFLA, AAs 345–351), found in the C-proximal part of PVA HC-Pro. Mutations in the 4E-binding site of HC-Pro reduce the virulence of PVA. This interaction may enhance the association of HC-Pro with eIF4E or its isoform in the virus replication/translation complex and thus increase viral RNA translation at the expense of cellular mRNA translation. Alternatively, these interactions may selectively inhibit host cell protein production. A comparison of the HC-Pro sequences of 47 potyviruses indicated that the hydrophobic Ala residue was fully conserved, and that Tyr and Leu were highly conserved. The TEV HC-Pro putative initiation factor 4E-binding site was among the outliers. In 28 TEV isolates, the C-proximal region of HC-Pro contained another motif (YLLSILY, residues 391–397), also corresponding to the consensus 4E-binding motif (YXXXXLΦ, where X is a variable amino acid, and Φ is a hydrophobic residue) [91]. The peanut stripe virus (PStV) HC-Pro also interacts with eIF4E and its isoform eIF(iso)4E [109].

### 4.4. Effects of HC-Pro on Methylation May Affect RNA Exonuclease Degradation

In plants, HC-Pro increases the amount of non-methylated small RNAs (sRNAs). sRNA stabilization requires methylation, so this interaction may weaken RNA silencing against potyviral infection [100]. In line with the above, inhibiting sRNA methylation can lead to sRNA degradation by exonucleases. As shown by Jamous et al. (2011), the zucchini yellow mosaic virus (ZYMV) HC-Pro interacts with the HEN1 (Hua Enhancer1) methyltransferase, inhibiting methylation [38]. The region responsible for this interaction is located between AAs 139 and 320. The TuMV HC-Pro inhibits HEN1 methylation activity in vitro and in vivo via its FRNK motif [39]. 

RNA methylation may also be regulated via the methionine cycle and inhibition of key enzymes that provide the substrates for methylation, namely S-adenosylmethionine synthase and S-adenosylhomocysteine hydrolase [37,100]. According to this general mechanism of methylation inhibition, HC-Pro may influence several other processes requiring methylation, protein methylation, and nucleotide synthesis. 

HC-Pro affects the methionine cycle in the cytoplasm, thus affecting RNA methylation, but not DNA methylation, which occurs in the nucleus [37,116]. Nevertheless, the tobacco vein banding mosaic virus (TVBMV) HC-Pro interferes with RNA-directed DNA methylation, decreases the DNA methylation of the promoters YUC1, YUC5, and YUC10, which directly activate the transcription of auxin biosynthesis genes, and can also activate the salicylic acid signaling pathway [110,111]. HC-Pro contains a domain that binds to dsDNA, and such binding does not require a specific DNA sequence or length [99]. 

HC-Pro binding to the nucleosome is based on interactions with histones, more specifically H3 and H4. HC-Pro’s interactions with histones H3 and H4 may prevent histone methylation, most likely directly promoting virus replication and inducing disease symptoms or even indirectly assisting in the inactivation of gene silencing, thereby countering the host defense system. Histone methylation is central to epigenetic modification regulated by methyltransferase activity [99]. Moreover, histone–lysine N-methyltransferase (EC 2.1.1.-) may be a substrate for proteolytic cleavage by HC-Pro (Tables S1 and S2).

### 4.5. HC-Pro’s Interactions with the Host Protein VARICOSE Play Several Roles in Various Stages of Infection and Are Associated with Gene Silencing Suppression

VARICOSE (VCS) belongs to a large family of eukaryotic proteins with WD40 domains. These domains consist of tandemly repeated stretches of approximately 40–60 AAs starting with glycine–histidine (GH) and ending with tryptophan–aspartate (WD). WD40 domains usually fold into a multiple β-propeller structure with an open-ended cylindrical shape and often serve as hubs of cellular networks. Thus, they play key roles in many biological processes, supporting the reversible assembly of multiprotein complexes [41]. 

In host cells, VCS is involved in seedling development and in the assembly of protein complexes associated with RNA metabolism. In the context of PVA infection, VCS is an important infectivity factor. The association of the PVA HC-Pro with the host protein VCS is crucial for RNA silencing suppression, potyvirus-induced RNA granule formation (structures involved in the protection of viral RNAs against the RNA-silencing machinery of the host), viral protein translation, virion stability, and systemic PVA infectivity [41]. 

De et al. (2020) proposed a hypothetical model for a multiprotein complex composed of HC-Pro, VCS, AGO1, eIF4A, and eIF(iso)4A. The composition of this complex likely varies with the stage of infection [41]. The conserved five-amino acid motif AELPR in the C-terminal region of the PVA HC-Pro has been identified as a putative interaction site for WD40 domain-containing proteins, including VRC [41]. 

### 4.6. HC-Pro’s Interactions with Calcium-Binding Proteins May Affect Host Defense

HC-Pro interacts with calcium-binding proteins. These interactions may be involved in plant signaling pathways and interfere with virus infection and host defense [97,102]. A calmodulin-related protein (termed the regulator of gene silencing calmodulin-like protein (rgs-CaM)) interacts with HC-Pro, and both rgs-CaM and HC-Pro suppress gene silencing [102]. This finding indicates that calcium regulates the activity of the gene-silencing pathway and that the downstream target proteins (subsequently activated by calmodulin and related proteins) of rgs-CaM may also be gene silencing suppressors [102]. 

Also, the host factor full-length calreticulin of *Carica papaya* L. plants specifically interacts with the papaya ringspot virus HC-Pro [97]. Deletion mutant studies have demonstrated that the calreticulin C-domain (residues 307–422), with a high Ca^2+^-binding capacity, is responsible for binding to the papaya ringspot virus HC-Pro. But, unlike the host defense suppressor rgs-CaM, calreticulin is significantly upregulated in the primary stage of infection and its increased expression may be an early defense response to viral infection in *Carica papaya* L. plants [97].

### 4.7. HC-Pro’s Interactions with Microtubule-Associated Proteins May Modulate Virus Infection

Some viruses may regulate host functions via the microtubule network. The viral regulation of microtubule dynamics and organization and virus-microtubule interactions influence virus movement and accumulation [103]. *Solanum tuberosum* L. and *Nicotiana tabacum* L. microtubule-associated proteins HIP1 and HIP2 (HIP: HC-Pro-interacting protein) interact with the PVA HC-Pro in PVA-infected plant cells [87,103,112]. HC-Pro interacts with HIP2 along microtubules, especially at microtubule junctions. Containing a stretch of six residues with a highly variable region in potyviruses, the C-proximal region of the PVA HC-Pro determines its interactions with HIP2, regulating the conformation of HC-Pro and virus–host interactions. Consequently, highly variable region mutations may induce host defense [87,103]. 

PVA accumulation in inoculated tissues was significantly reduced in HIP2-silenced leaves of *Nicotiana benthamiana*, delaying systemic PVA infection. This result indicates that HIP2–HC-Pro interactions are important for virus infection [87,103]. However, HIP2–HC-Pro interactions may not be directly involved in viral replication but may help viruses control host responses and hence modulate the cellular environment for their benefit. Similarly to the HIP2 analog of *Arabidopsis thaliana*, HC-Pro may interact with the proteins involved in host antiviral defense signaling [87,103].

### 4.8. HC-Pro Interacts with Host Enzymes

In addition to Rubisco, methyltransferase, and key enzymes of the methionine cycle discussed above, HC-Pro interacts with other important host enzymes. The sugarcane mosaic virus (SCMV) HC-Pro interacts with *Zea mays* L. triacylglycerol lipase (TGL), reducing virus accumulation in these plants [98]. Degradation caused by the proteasome or autophagy is not responsible for reduced HC-Pro levels, but, surprisingly, the hydrolase activity of lipases is responsible. Mutating amino acids essential for TGL catalysis (S161A/D255A/H304A) offset the effects of reduced virus accumulation, so the infection reaches its usual extent. In addition, TGL activates the salicylic acid signaling pathway, which inhibits SCMV infection [98]. 

SCMV infection attenuation and HC-Pro RNA silencing suppression also result from a specific interaction between *Zea mays* L. violaxanthin deepoxidase (Zm VDE) and SCMV HC-Pro [96]. AAs 101–460 in HC-Pro and AA Q 292 in ZmVDE are essential for this interaction. VDE is a key enzyme in the xanthophyll cycle, catalyzing the conversion of violaxanthin into zeaxanthin under excessive-light conditions and thus protecting the photosynthetic apparatus from photodamage and photoinhibition. However, photoinhibition is also induced by abiotic stress, and VDE has been recently shown to respond to biotic stress. Potentially attenuating SCMV HC-Pro-induced RNA silencing suppression, ZmVDE may be involved in a secondary defense response against viral infection in monocot plants. Furthermore, VDE may relocalize to HC-Pro-containing aggregation bodies, and its expression is induced in eIFiso4G-knockout *A. thaliana* plants [96,117]. 

The PVY HC-Pro interacts with the host tobacco protein 1-deoxy-D-xylulose-5-phosphate synthase (NtDXS), which promotes the biosynthesis of plastidic isoprenoids, as shown in plants infected with PVY [95]. DXS catalyzes the rate-limiting step of the plastidic 2-C-methyl-D-erythritol-4-phosphate (MEP) pathway, which produces isopentenyl diphosphate as a precursor for the synthesis of many isoprenoids, such as chlorophylls, carotenoids, and abscisic acid. Based on in vitro assays, HC-Pro increases DXS activity. DXS contains a thiamin pyrophosphate-binding domain that can bind to the N-terminal domain of HC-Pro (residues 1–97). This binding leads to a conformational change in the enzyme structure, thereby decreasing the activation energy and increasing the reaction rate [95].

Potyviral TuMV HC-Pro can interact with *Chenopodium quinoa* (Willd.) carbonic anhydrase 1 (CA 1) and its *A. thaliana* homolog AtCA1, which has been described as the salicylic acid-binding protein 3. The interaction of the regulator AtCA1 with HC-Pro compromises the salicylic acid pathway, weakening host defense responses and facilitating viral infection. These findings highlight a new opportunity for modulating innate immunity [104]. 

Potyviral infection causes many changes in plant physiology, including reduced photosynthesis and, conversely, activated defense reactions and phenolics synthesis, and increased antioxidant enzyme activity [118]. Infection with PVY alters host plant metabolism, increasing metabolite flux through anaplerotic pathways and phosphoenolpyruvate carboxylase (PEPC) and NADP-malic enzyme activity, thereby providing the plant with NADPH at the expense of ATP and simultaneously supplying CO_2_, which is otherwise unavailable as stomata are closed during viral infection [119]. The viral proteins involved in these metabolic changes have not been identified yet, but increased PEPC and NADP-malic enzyme activity is associated with PEPC phosphorylation, whereas increased NADP-malic enzyme activity is accompanied by increased protein expression and increased mRNA transcription of the cytosolic rather than the chloroplast isoform [120,121]. Nevertheless, HC-Pro is unlikely involved in these changes because no differences were found between transgenic plants carrying the PVA HC-Pro gene and wild-type plants [122].

### 4.9. HC-Pro Interacts with Antiviral Compounds

The PVY HC-Pro C-terminally truncated recombinant protein (residues 307–465) interacts with diaryl urea derivatives. In urea derivatives, the phenyl-N, NH-, and CO- functional groups form major binding categories. Molecular docking studies have identified Asp 121, Asn 48, and Tyr 38 as the sites of interaction between the PVY HC-Pro C-terminal domain and these compounds. Accordingly, these AA residues are important for the design and synthesis of novel urea derivatives as antiviral compounds [22]. 

## 5. Conclusions

This review summarizes the best-characterized functional structural motifs of HC-Pro responsible for aphid transmission, genome amplification, viral polyprotein autoproteolytic cleavage, cell-to-cell and long-distance movement, symptom development, and RNA silencing suppression. Structurally, the HC-Pro catalytic domain is a cysteine-type protease much smaller than papain, albeit with a similar active site, consisting of the catalytic dyad Cys and His. This domain autoproteolytically cleaves the potyviral polyprotein in the sequence motif YXVG/G, separating HC-Pro from the potyviral protein P3. Although 146 plant proteins of *Viridiplantae* containing the YXVG/G sequence have been identified in the UniProtKB database as potential HC-Pro substrates, no protein other than a viral polyprotein has been reported as a protease degradation target yet. The cleaved product may bind to the active cleft, inhibiting HC-Pro, but the corresponding mechanism and conditions under which HC-Pro may cleave host proteins remain unknown. Nevertheless, this review highlights key HC-Pro interactions with host proteins. Such interactions may determine whether host proteins enable or overcome viral infection. HC-Pro appears to be a very good servant of its master: most of HC-Pro’s interactions with host proteins (such as the 20S proteasome, methyltransferase, transcription factor eIF4E, salicylic acid-binding protein 3, and microtubule-associated protein HIP2) facilitate the spread of viral infection and modulate the cellular environment, promoting virus accumulation, exacerbating symptom severity (interactions with MinD, Rubisco, and ferredoxin) and suppressing RNA silencing (interactions with host proteins VARICOSE and the calmodulin-like protein). In contrast, HC-Pro’s interactions with some plant enzymes—be it TGL, which directly cleaves HC-Pro, or the enzyme that promotes isoprenoid biosynthesis—seem to be beneficial for the host plant. The strength of these interactions between HC-Pro and the corresponding proteins varies with the plant species, so these interactions may contribute to the sensitivity of a given plant species to potyviruses.

## Figures and Tables

**Table 1:** HC-Pro and its plant host interaction partners and processes.

Interaction Partner	Plant	HC-Pro Origin [Reference]
**20S proteasome subunits:**		
PAA (α 1) PBB (β 2) PBE (β 5)	*Arabidopsis thaliana* L.	Potato virus Y [63] (PVY)
PAE (α 5)	*Arabidopsis thaliana* L.	Lettuce mosaic virus [94] (LMV)
**Related to autophagy:**		
Autophagy cargo receptor NBR1	*Nicotiana benthamiana*	Turnip mosaic virus (TuMV) [106]
**Chloroplastic proteins**:		
Ca^2+^-dependent ATPase (MinD)	*Nicotiana tabacum* L. *Nicotiana benthamiana*	Potato virus Y (PVY) [64]
ATP-synthase NtCF1β-subunit	*Nicotiana tabacum* L.	Potato virus Y (PVY) [101,107]
Rubisco	*Phaseolus vulgaris* L.	Bean common mosaic virus (BCMV) [99]
Ferredoxin-5 (Fd V)	*Zea mays* L.	Sugarcane mosaic virus (SCMV) [62]
**Transcription and translation factors:**		
Ethylene-inducible transcription factor	*Nicotiana tabacum* L. *Arabidopsis thaliana* L.	Turnip mosaic virus (TuMV) [108]
Translation initiation factor eIF4E and its isoform eIF(iso)4E	*Nicotiana tabacum* L.	Potato virus Y, A (PVY, PVA) Tobacco etch virus (TEV) [91]
Translation initiation factor eIF4E and its isoform eIF(iso)4E	*Arachis hypogaea* L.	Peanut stripe virus (PStV) [109]
**Related to methylation:**		
HEN 1 methyltransferase	in vitro	Zucchini yellow mosaic virus (ZYMV) [38]
HEN 1 methyltransferase	*Arabidopsis thaliana* L. *Marchantia polymorpha* L.	Turnip mosaic virus (TuMV) [39]
Methionine cycle key enzymes	*Nicotiana benthamiana*	Potato virus A (PVA) [37]
Histones H3, H4 Nucleosome	*-*	Bean common mosaic virus (BCMV) [99]
DNA methylation of the promoters of YUC genes affecting auxin biosynthesis	*Arabidopsis thaliana* L.	Tobacco vein banding mosaic virus (TVBMV) [110]
DNA methylation of the promoters of the regulators in the salicylic acid pathway	*Arabidopsis thaliana* L.	Tobacco vein banding mosaic virus (TVBMV) [111]
**Related to gene silencing:**		
ARGONAUTE1	*Nicotiana benthamiana*	Potato virus A (PVA) [3]
Host protein VARICOSE	*Nicotiana benthamiana*	Potato virus A (PVA) [41]
**Related to calcium:**		
Calmodulin-related protein (rgs-CaM)	*Nicotiana tabacum* L. *Nicotiana benthamiana*	Tobacco etch virus (TEV) [102]
Calreticulin	*Carica papaya* L.	Papaya ringspot virus (PRSV) [97]
**Related to microtubules**:		
Microtubule-associated host protein HIP2	*Solanum tuberosum* L. *Nicotiana tabacum* L. *Nicotiana benthamiana*	Potato virus A (PVA), Tobacco etch virus (TEV) [87,103]
Novel RING finger protein HIP1	*Solanum tuberosum* L.	Potato virus A (PVA) [112]
**Enzymes:**		
Triacylglycerol lipase	*Zea mays* L.	Sugarcane mosaic virus (SCMV) [98]
Violaxanthin deepoxidase	*Zea mays* L.	Sugarcane mosaic virus (SCMV) [96]
1-deoxy-D-xylulose-5-phosphate synthase	*Nicotiana benthamiana*	Potato virus Y (PVYN) [95]
Carbonic anhydrase (salicylic acid-binding protein 3)	*Arabidopsis thaliana* L.	Turnip mosaic virus (TuMV) [104]
**Urea derivatives**	in vitro	Potato virus Y (PVY) [22]

## Data Availability

All data are contained within the article or Supplementary Materials. The raw data are available on request from the corresponding author.

## Supplementary Materials

The following supporting information can be downloaded at https://www.mdpi.com/article/10.3390/plants13091236/s1, Table S1: All proteins containing the YxVGG sequence motif potentially cleaved by HC-Pro in the *Viridiplantae* searched in the UniProtKB database using the PROSITE. Table S2: Selected proteins containing the YxVGG sequence motif potentially cleaved by HC-Pro.
