# Supplementary material for: Potyviral Helper-Component Protease: Multifaced Functions and Interactions with Host Proteins
Source: Plants (Basel). 2024 Apr 29;13(9):1236. doi: 10.3390/plants13091236 (PMC11085613; doi:10.3390/plants13091236)
Supplement: Supplementary file 1 [file plants-13-01236-s001.zip › Table S2.pdf]

Table S2. Selected proteins containing the YxVGG sequence motif that could be possibly cleaved by HC-Pro. The role in viral infection shows a possibility how the plant protein cleavage by HC-Pro could help the virus to spread or to influence the plant metabolism.

<table>
<tr><th>Plant protein</th><th>Plant</th><th>ID</th><th>Sequence</th><th>Role in viral infection</th></tr>
<tr><td>ATP synthase subunit β, mitochondrial (EC 7.1.2.2)</td><td>Actinidia deliciosa (Kiwi)</td><td>>sp|P43395|ATPBM_ACTDE (173 aa)</td><td>152 - 156: YmVGG</td><td rowspan="9">In apple plants, the mitochondrial ATP synthase oligomycin sensitivity-conferring protein subunit interacts with apple necrotic mosaic virus coat protein and facilitates its infection [1]. In chloroplasts, ATP synthase-α plays active roles in plant defense. Tobacco mosaic virus (TMV) infection reduced the expression levels of the ATP synthase-γ gene and silencing ATPsyn-γ in Nicotiana benthamiana increased TMV accumulation and pathogenicity [2]. HC-Pro was shown to decrease the amount of ATP-synthase CF1 β-subunit in PVY-infected plants [3,4] and also to interact with Ca^{2+}-dependent ATP-synthase, MinD [5].</td></tr>
<tr><td>ATP synthase subunit β-1, mitochondrial (EC 7.1.2.2)</td><td>Arabidopsis thaliana</td><td>>sp|P83483|ATPBM_ARATH (556 aa)</td><td>535 - 539: YmVGG</td></tr>
<tr><td>ATP synthase subunit β, mitochondrial (EC 7.1.2.2)</td><td>Chlamydomonas reinhardtii (Chlamydomonas smithii)</td><td>>sp|P38482|ATPBM_CHLRE (574 aa)</td><td>487 - 491: YmVGG</td></tr>
<tr><td>ATP synthase subunit β, mitochondrial (EC 7.1.2.2)</td><td>Hevea brasiliensis (Siphonia brasiliensis)</td><td>>sp|P29685|ATPBM_HEVBR (562 aa)</td><td>541 - 545: YmVGG</td></tr>
<tr><td>ATP synthase subunit β, mitochondrial (EC 7.1.2.2)</td><td>Zea mays)</td><td>>sp|P19023|ATPBM_MAIZE (553 aa)</td><td>532 - 536: YmVGG</td></tr>
<tr><td>ATP synthase subunit β, mitochondrial (EC 7.1.2.2)</td><td>Nicotiana plumbaginifolia</td><td>>sp|P17614|ATPBM_NICPL (560 aa)</td><td>539 - 543: YmVGG</td></tr>
<tr><td>ATP synthase subunit β, mitochondrial (EC 7.1.2.2).</td><td>Oryza sativa subsp. japonica</td><td>>sp|Q01859|ATPBM_ORYSJ (552 aa)</td><td>531 - 535: YmVGG</td></tr>
<tr><td>ATP synthase subunit β-2, mitochondrial (EC 7.1.2.2)</td><td>Arabidopsis thaliana</td><td>>sp|P83484|ATPBN_ARATH (556 aa)</td><td>535 - 539: YmVGG</td></tr>
<tr><td>ATP synthase subunit β-3, mitochondrial (EC 7.1.2.2)</td><td>Arabidopsis thaliana</td><td>>sp|Q9C5A9|ATPBO_ARATH (559 aa)</td><td>538 - 542: YmVGG</td></tr>
<tr><td>Chlorophyll a-b binding protein 215, chloroplastic (LHCII type II CAB-215) (LHCP)</td><td>Pisum sativum</td><td>>sp|P27520|CB215_PEA (265 aa)</td><td>174 - 178: YrVGG</td><td rowspan="5">The photosynthetic rate of virus-infected plants is always reduced [4] Damaged photosynthetic complexes and degraded chloroplasts are related to plant viral infection [6].</td></tr>
<tr><td>Chlorophyll a-b binding protein 151, chloroplastic (LHCII type II CAB-151) (LHCP)</td><td>Gossypium hirsutum (Gossypium mexicanum)</td><td>>sp|P27518|CB21_GOSHI (265 aa)</td><td>174 - 178: YrVGG</td></tr>
<tr><td>Chlorophyll a-b binding protein of LHCII type I, chloroplastic (CAB) (LHCP)</td><td>Lemna gibba</td><td>>sp|P12328|CB21_LEMGI (264 aa)</td><td>173 - 177: YrVGG</td></tr>
<tr><td>Chlorophyll a-b binding protein type I, chloroplastic (CAB) (LHCP)</td><td>Pinus thunbergii (Pinus thunbergiana)</td><td>>sp|P10049|CB21_PINTH (266 aa)</td><td>175 - 179: YrVGG</td></tr>
<tr><td>Chlorophyll a-b binding protein, chloroplastic</td><td>Silene latifolia subsp. alba (Lychnis alba)</td><td>>sp|P12332|CB21_SILLB (205 aa)</td><td>173 - 177: YrVGG</td></tr>
</table>

<table>
<tr><td>(LHCII type I CAB) (LHCP).</td><td></td><td></td><td></td><td></td></tr>
<tr><td>Chlorophyll a-b binding protein type 2 member 2 (Chlorophyll a-b binding protein type II 2) (CAB) (LHCP)</td><td>Pinus sylvestris</td><td>>sp|P15192|CB22_PINSY (150 aa)</td><td>59 - 63: YrVGG</td><td></td></tr>
<tr><td>Chlorophyll a-b binding protein, chloroplastic (LHCII type I CAB) (LHCP)</td><td>Oryza sativa subsp. indica</td><td>>sp|A2XJ35|CB23_ORYSI (263 aa)</td><td>172 - 176: YrVGG</td><td></td></tr>
<tr><td>Chlorophyll a-b binding protein, chloroplastic (LHCII type I CAB) (LHCP)</td><td>Oryza sativa subsp. japonica</td><td>>sp|Q10HD0|CB23_ORYSJ (263 aa)</td><td>172 - 176: YrVGG</td><td></td></tr>
<tr><td>Chlorophyll a-b binding protein 36, chloroplastic (LHCII type I CAB-36) (LHCP)</td><td>Nicotiana tabacum</td><td>>sp|P27494|CB23_TOBAC (265 aa)</td><td>174 - 178: YrVGG</td><td></td></tr>
<tr><td>Chlorophyll a-b binding protein 4, chloroplastic (LHCII type I CAB-4) (LHCP)</td><td>Solanum lycopersicum (Lycopersicon esculentum)</td><td>>sp|P14278|CB24_SOLLC (265 aa)</td><td>174 - 178: YrVGG</td><td></td></tr>
<tr><td>Chlorophyll a-b binding protein 5, chloroplastic (LHCII type I CAB-5) (LHCP)</td><td>Solanum lycopersicum (Lycopersicon esculentum)</td><td>>sp|P14279|CB25_SOLLC (237 aa)</td><td>146 - 150: YrVGG</td><td></td></tr>
<tr><td>Chlorophyll a-b binding protein 37, chloroplastic (LHCII type I CAB-37) (LHCP)</td><td>Petunia sp.</td><td>>sp|P12062|CB26_PETSP (265 aa)</td><td>174 - 178: YrVGG</td><td></td></tr>
<tr><td>Cysteine--tRNA ligase CPS1, chloroplastic/mitochondrial (EC 6.1.1.16) (Chloroplast synthesis protein 1) (Cysteinyl-tRNA synthetase)</td><td>Zea mays L.</td><td>>sp|A0A1D6LAG9|CPS1_MAIZE (564 aa)</td><td>200 - 204: YvVGG</td><td>The chloroplastic isoform of the plant tRNA ligase mediates the replication of the viroids belonging to the family Avsunviroidae [7].</td></tr>
<tr><td>Endoribonuclease Dicer homolog 3b (Dicer-like protein 3b) (OsDCL3b) (EC 3.1.26.-)</td><td>Oryza sativa subsp. japonica</td><td>>sp|Q7XD96|DCL3B_ORYSJ (1637 aa)</td><td>1195 - 1199: YyVGG</td><td>Dicer-like proteins are the central enzymes of RNA silencing which regulate gene expression and represent a way of defense against viruses [8].</td></tr>
<tr><td>ToMV resistance protein Tm-1(GCR237) (Disease resistance protein Tm-1) (Protein p80(GCR237))</td><td>Solanum lycopersicum (Lycopersicon esculentum)</td><td>>sp|A7M6E7|TM1R_SOLLC (754 aa)</td><td>276 - 280: YvVGG</td><td rowspan="2">Tm-1 resistance protein binds ToMV replication proteins and inhibits the RNA-dependent RNA replication of ToMV [9].</td></tr>
<tr><td>ToMV susceptible protein tm-1(GCR26) (Disease susceptible protein tm-1) (Protein p80(GCR26))</td><td>Solanum lycopersicum (Lycopersicon esculentum)</td><td>>sp|A7M6E8|TM1S_SOLLC (754 aa)</td><td>276 - 280: YvVGG</td></tr>
</table>

Disease resistance protein RPV1 (NAD(+) hydrolase RPV1) (EC 3.2.2.6) (Resistance to Plasmopara viticola protein) (MrRVP1)	*Vitis rotundifolia*	>sp|V9M2S5|RPV1_VITRO (1398 aa)	223 - 227: YgVGG	Disease resistance proteins RPV1 and RUN1 mediate NAD^+ cleavage that triggers a defense system that promotes programed cell death as shown against fungal pathogens [10,11].
Disease resistance protein RUN1 (NAD($^+$) hydrolase RUN1) (EC 3.2.2.6) (NADP($^+$) hydrolase RUN1) (EC 3.2.2.-) (Resistance to Uncinula necator protein) (MrRUN1)	*Vitis rotundifolia*	>sp|V9M398|RUN1_VITRO (1331 aa)	228 - 232: YgVGG	
G-type lectin S-receptor-like serine/threonine-protein kinase At1g11280 (EC 2.7.11.1)	*Arabidopsis thaliana*	>sp|Q9SXB3|Y1112_ARATH (820 aa)	411 - 415: YsVGG	Role of lectins in plant defense is not entirely elucidated. The lectin gene Restricted TEV movement 1 inhibits the systemic movement of potyviruses and Jacalin-Type lectin required for potexvirus resistance 1 impairs the accumulation of plantago asiatica mosaic virus (PlAMV) RNA at the cellular level [12].
L-type lectin-domain containing receptor kinase IX.2 (LecRK-IX.2) (EC 2.7.11.1)	*Arabidopsis thaliana*	>sp|Q9LSL5|LRK92_ARATH (675 aa)	132 - 136: YsVGG	
Probable LRR receptor-like serine/threonine-protein kinase At3g47570 (EC 2.7.11.1)	*Arabidopsis thaliana*	>sp|C0LGP4|Y3475_ARATH (1010 aa)	892 - 896: YgVGG	
Polyamine oxidase 6 (OsPAO6) (EC 1.5.3.-) (Polyamine oxidase B)	*Oryza sativa* subsp. *japonica*	>sp|A0A0P0XM10|PAO6_ORYSJ (496 aa)	489 - 493: YnVGG	Plants exhibit increased polyamine levels during viral infection, which are rapidly degraded by polyamine oxidases, which are also upregulated upon virus infection [13]. The increase of polyphenol oxidase activity was found in tobacco plants infected with tobacco mosaic virus (TMV) [14].
Polyphenol oxidase E, chloroplastic (PPO) (EC 1.10.3.1) (Catechol oxidase)	*Solanum lycopersicum* (*Lycopersicon esculentum*)	>sp|Q08307|PPOE_SOLLC (587 aa)	187 - 191: YkVGG	
Catechol oxidase B, chloroplastic (EC 1.10.3.1) (Polyphenol oxidase) (PPO)	*Solanum tuberosum*	>sp|Q06355|PPOB_SOLTU (588 aa)	188 - 192: YkVGG	
WAT1-related protein At2g37460	*Arabidopsis thaliana*	>sp|Q9ZUS1|WTR13_ARATH (380 aa)	268 - 272: YyVGG	The protein Walls are thin 1 (WAT1), a plant-specific protein that dictates secondary cell wall thickness of wood fibres and was found to facilitate auxin transport in Arabidopsis [15].
Histone-lysine N-methyltransferase ATX2 (EC 2.1.1.-) (Protein SET DOMAIN GROUP 30) (Trithorax-homolog	*Arabidopsis thaliana*	>sp|P0CB22|ATX2_ARATH (1083 aa)	873 - 877: YiVGG	The interaction between HC-Pro from turnip mosaic virus (TuMV) or zucchini yellow mosaic virus (ZYMV) and Hua Enhancer1 (HEN1) led to

protein 2) (TRX-homolog protein 2)	inhibition of methyltransferase activity [16,17].
